# Investment in Safe Routes to School Projects: Public Health Benefits for the Larger Community

**Published:** 2008-06-15

**Authors:** Margaret Watson, Andrew Dannenberg

**Affiliations:** Oak Ridge Institute for Science and Education Fellow, Division of Cancer Prevention and Control, CDC. Ms Watson conducted this research while fulfilling the practicum requirement for the Master of Public Health degree at the Institute of Public Health, Georgia State University; Division of Emergency and Environmental Health Services, National Center for Environmental Health, CDC, Atlanta, Georgia

## Abstract

**Introduction:**

The Safe Routes to School (SRTS) program is designed to encourage active and safe transportation for children to school. This report examines the potential broader impact of these programs on communities within 0.5 mile (0.8 km) of schools.

**Methods:**

We used a geographic information system to generate estimates of the land area within 0.5 mile of public schools in 4 U.S. Census-defined categories: 37 large urban areas, 428 small urban areas, 1088 metropolitan counties (counties in metropolitan statistical areas excluding the urban areas), and 2048 nonmetropolitan counties. We estimated population at the county level or at the U.S. Census-defined urban-area level using data from the 2000 U.S. Census.

**Results:**

In large urban areas, 39.0% of the land area was within 0.5 mile of a public school, and in small urban areas, 26.5% of the land area was within 0.5 mile of a public school. An estimated 65.5 million people in urban areas could benefit from SRTS projects. In nonurban areas, 1% or less of land is within 0.5 mile of a public school.

**Conclusion:**

Results suggest that SRTS projects in urban areas can improve the walking and bicycling environment for adults as well as for children, the target users. Investment in SRTS can contribute to increased physical activity among children and adults.

## Introduction

In 2005, the federal Safe, Accountable, Flexible, Efficient Transportation Equity Act: A Legacy for Users established a national Safe Routes to School (SRTS) program. From 2005 through 2009, $612 million in federal transportation funds will be made available to state, local, and regional agencies and to nonprofit organizations for programs that encourage primary and middle school students to walk or bike to school. Programs must use at least 70% but no more than 90% of the funds on infrastructure-related projects, which may include sidewalk improvements, traffic-calming measures, bicycle lanes, and bike racks (1). Noninfrastructure-related projects may include student and parent education, public awareness campaigns, and traffic enforcement (2).

Improving the physical infrastructure of neighborhoods may encourage children (and parents accompanying them) to walk or bike to school. Physical activity has been consistently shown to improve health (3). People are more likely to be physically active if they have nearby places to participate in physical activity and if urban design and land use practices at the local level are conducive to physical activity (4). SRTS projects, such as improvements to sidewalks and crosswalks and construction of walking and bicycle trails, can provide substantial benefits to schoolchildren and the community. This study provides estimates of the areas and of the populations that could be affected by improvements in the streetscape through the SRTS program.

## Methods

We used a geographic information system (GIS) (ArcMap 9.0, ESRI, Redlands, California) to calculate estimates of the areas and populations that could be affected by SRTS funding. The primary spatial data sources were geographic boundaries and area characteristics from the 2000 U.S. Census (5) and a 2003–2004 database of elementary, middle, and high schools in the United States coded by geographic location (6).

We assigned land area in the United States to 1 of 4 categories: large urban areas, defined as having a population of at least 1 million; small urban areas, defined as having a population of 50,000–999,999; metropolitan counties (excluding urban areas); and nonmetropolitan counties. Urban areas are defined by the U.S. Census Bureau as being densely settled territories containing more than 50,000 people, with core census block groups or blocks that have a population density of at least 1000 people per square mile, and with surrounding census blocks that have an overall density of at least 500 people per square mile (7). We imported into the GIS a data file containing boundaries and other information about U.S. Census-defined urban areas, last updated in 2000.

We then classified land areas outside of urban areas as being in metropolitan statistical areas (MSAs) or outside of MSAs, which are defined at the county level. Using information from the U.S. Census Bureau, the federal Office of Management and Budget (OMB) defines MSAs as places associated with an urban area of more than 50,000 people and with a high degree of integration within the urban core (8). Note that we did not count urban clusters, defined as urban areas having fewer than 50,000 people, as urban areas. Urban clusters such as Curtis, Nebraska, are small towns in rural areas and are not associated with an MSA. We included small rural town areas in the nonmetropolitan areas category.

To categorize places outside of urban areas as metropolitan or nonmetropolitan, we imported a file containing information on U.S. counties into the GIS and joined it to a file containing 2003 rural-urban continuum codes (RUCCs). These codes, based on the OMB definition for MSAs, are available for every county in the United States on the U.S. Department of Agriculture Web site (9). We consolidated the original 9 RUCCs into 2 codes: counties in metropolitan areas and counties not in metropolitan areas. This consolidation resulted in 1088 metropolitan counties and 2048 nonmetropolitan counties. We counted only the portions of metropolitan counties outside of urban areas as metropolitan areas; portions of counties within urban areas were counted as urban areas. Therefore, a metropolitan county may be divided in these analyses as a part-urban area and a part-metropolitan area outside an urban area.

Finally, we imported into the GIS a data file, or layer, for 2003–2004 that contained the location and other information about U.S. public schools with grades pre-kindergarten through 12th (6). Approximately 90% of children in the United States attend public schools (10). We did not include private and parochial schools in the study because data for them are less available and less reliable. In addition, we excluded 14,675 special education schools, vocational schools, other/alternative schools, and schools having no students (such as new schools that are not yet operational or schools in the process of closing). The remaining 85,919 schools were then classified into 4 mutually exclusive categories: 25,938 were in large urban areas, 19,740 were in small urban areas, 16,142 were in metropolitan counties (outside of an urban area), and 24,099 were in nonmetropolitan counties.

To analyze the area and population potentially affected by SRTS programs, we created buffers with a radius of 0.5 mile around a point at the center of each school. These buffers counted only the area within 0.5 mile of a school and did not take into account the actual distance to a school by the road network or the connectivity of roads or paths. One mile is considered a reasonable distance to walk; 2 miles is considered a reasonable distance to bike (11). Students living within 1 mile of school are more likely to walk to school than are students living farther away (12). However, we were not measuring distance along road networks, so we chose 0.5-mile buffers for this analysis because distance along the road network is generally longer than a direct route. Those living within the 0.5-mile buffer are more likely to be close enough to walk than are those living farther away. Given that only 31% of trips made to school by students living within 1 mile of school in 1995 were made by walking (11), it is unlikely that many of those living farther than 1 mile would choose to walk. The *Healthy People 2010* goal that relates to walking to school aims to increase the number of walkers living within 1 mile of a school, but this distance is measured by self-reports (11). We assumed that most people reported the distance by measuring along streets and roads. Using 1-mile buffers could include many people who, in fact, live more than 1 mile away from the school when actual routes along streets and roads are measured. If 2 or more schools within a county or urban area had overlapping half-mile buffers, we combined these buffers to prevent double counting of any area or population. The total land area covered by the half-mile buffer around all public schools within an urban area or county was calculated. This figure was then divided by the total land area within each urban area or county to calculate the percentage of the total land area within 0.5 mile of the local schools. To estimate the total population potentially affected, the percentage of land area was multiplied by the total population of the county or urban area.

## Results

**Figure 1.**
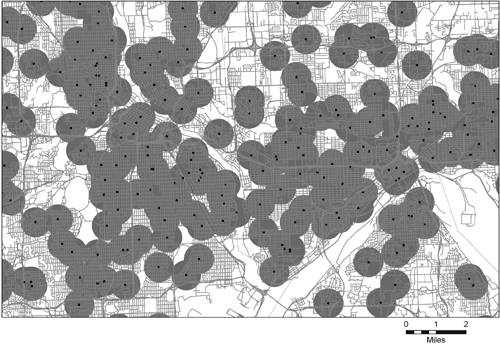
Example of school buffers of 0.5 mile in an urban area — Minneapolis-St. Paul, Minnesota (population: 2,388,593).

**Figure 2.**
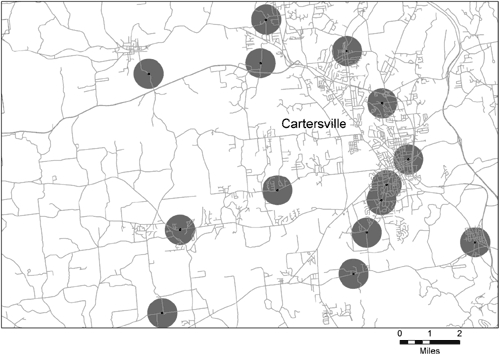
Example of school buffers of 0.5 mile in a metropolitan area outside an urban area — Bartow County, Georgia (county population: 76,019).

**Figure 3.**
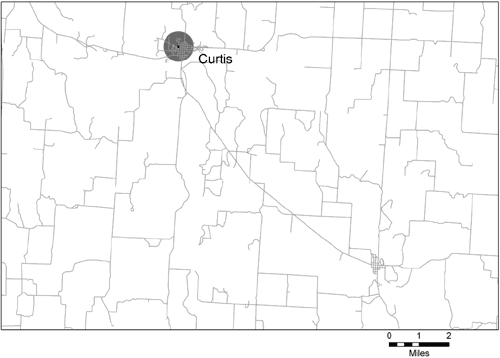
Example of school buffers of 0.5 mile in a nonmetropolitan area — Frontier County, Nebraska (county population: 3099).

The proportion of land area covered by the half-mile school buffers varies considerably by type of location. Examples of maps from each type of area are in Figure 1 (urban area), Figure 2 (metropolitan area outside an urban area), and Figure 3 (nonmetropolitan area). We chose these areas because they appeared to be visually representative of each type of area. All 3 maps use the same scale. In Figure 1, the map of the Minneapolis-St. Paul, Minnesota, urban area shows that half-mile school buffers cover most of the area, and most of the buffers overlap at least a portion of another buffer. Figure 2 shows Bartow County, Georgia, a metropolitan county outside of the urban area of Atlanta. Few of the buffers in this map are connected. Schools are mostly on the edge of, but not in, areas of high road network density. Figure 3 shows rural Frontier County, Nebraska; only 1 buffer appears on this map. The school is in the middle of the largest town on the map, and the buffer almost entirely covers the town's network of streets. The smaller pattern of streets several miles away is a small town with no local school.

### National findings

In the 37 large urban areas, 39.0% of the land area was within 0.5 mile of a public school (Table), and in the 428 small urban areas, 26.5% of the land area was within 0.5 mile of a public school. In the metropolitan counties outside of the U.S. Census-defined urban areas, only 1% of the land was within 0.5 mile of a public school. Less than 1% of the land area (0.5%) in nonmetropolitan counties was within 0.5 mile of a public school. We counted 25,938 public schools in large urban areas and 24,099 public schools in nonmetropolitan areas. Although these numbers are similar, the difference in total land area (34,040 square miles vs 2,680,545 square miles) means that a greater proportion of land in large urban areas is within 0.5 mile of a public school.

Assuming that population is distributed evenly in each urban area, at least 65 million people living in urban areas live within 0.5 mile of a public school. Approximately 45 million people are in large urban areas, and 20 million are in small urban areas. However, population is not distributed evenly, and areas that are more populous have more schools. In nonurban areas, population density is likely to be more varied, and county-level estimates do not capture the density variation within the county. Estimating population using units more precise than counties and metropolitan areas is beyond the scope of this research.

### A state example — Georgia

We conducted a separate analysis for the state of Georgia by using general methods similar to those above, except that the half-mile school buffers were combined with census blocks to determine more specific estimates of the Georgia population that would be affected by SRTS programs. Our analysis showed that approximately 1,367,000 people in the large and small urban areas live within 0.5 mile of a school in Georgia. In metropolitan areas outside of urban areas, approximately 113,000 people live within 0.5 mile of a school, and in rural areas the number is approximately 187,000. These numbers translate to 26% of the population in urban areas, 7% in metropolitan areas and 11% in rural areas. In other words, Georgia residents are twice as likely to live near a public school in urban areas as in metropolitan or rural areas. Statewide, approximately 20% of Georgia's 8.1 million people live within 0.5 mile of a public school. Percentages were probably higher in rural areas than in metropolitan areas because in many rural areas the population tends to be concentrated in small towns, whereas in metropolitan areas the population is more evenly distributed.

In our definitions of urban and rural areas, towns with fewer than 50,000 residents were not counted as being urban. However, many of these smaller towns have town centers, and substantial numbers of people may live within walking distance of a school. For example, of the 38,407 people who live in rural Tift County, Georgia, approximately 12,530 live within 0.5 mile of a school, mostly in the county seat of Tifton. Our calculations in Georgia were conducted at the census-block level; achieving such detail at the national scale is beyond the scope of this research. More detailed studies are needed to determine how many people in rural areas could be affected by improvements in walkability.

## Discussion

Increasing the number of children who walk and bicycle to school is one way to encourage physical activity and improve child health. From 1969 through 2001, the number of trips made by walking for children living within 1 mile of school declined from 87% to 36% (13,14). The Centers for Disease Control and Prevention (CDC), through its *Healthy People 2010* goals, aims to increase this number to 50% by 2010 (14). Active commuting to school can provide an opportunity for children to engage in physical activity during school days. Evidence shows that SRTS programs increase the number of children who get to school by active transportation and that the programs make the trip to and from school safer for those already walking (15-17).

During the past 30 years, rates of obesity and numbers of overweight children and adults have drastically increased (18). Although the link between the built environment and obesity is not as well established as the link with physical activity, increasing active commuting to school can increase physical activity and could affect rates of obesity and overweight. Given the high rates of childhood obesity and the consequences of adult obesity such as diabetes and heart disease, efforts should be made to encourage physical activity among children and adults.

Through the SRTS program, improving the walking areas around schools, especially in urban areas, could improve the entire community over time. We found that making such improvements in large and small urban areas could affect an estimated 65 million Americans. Research has documented that residents of more "walkable" neighborhoods, which have sidewalks and connected streets, walk more (19). Therefore, increasing neighborhood walkability may affect people in the larger community, not just schoolchildren. Walking can reduce rates of overweight, obesity, and diabetes, and people living in areas in which it is convenient to walk are more likely to do so (20-24). Recent research indicates that walkable neighborhoods may also contribute to better air quality and encourage the development of social capital among residents (25-27).

Improvements in the built environment, however, are not always possible to accomplish given budgetary limitations. SRTS programs were conceived to improve safety and to increase active transportation to school for students. If used as intended, such programs may also be an opportunity to make improvements to the streetscape of urban schools where improvements are neither politically nor economically feasible. Unfortunately, state regulations often encourage the placement of new schools on large swaths of land on the outskirts of cities and towns (27). Such school placement can decrease the number of people living within walking distance of a school (27).

### Limitations

Our study has several limitations. First, no standard definitions exist for urban, suburban, and rural areas. Although a number of different federal classifications by county are available, most such schemes assign the same area classification to all counties within an MSA, with no distinction between urban and suburban areas. Urban areas do not conform to county lines. For example, in Fulton County, Georgia, density by census tract ranges from 89 to 36,503 people per square mile. Second, our population estimates assume that the population in each urban area and county is distributed evenly. However, this is not the case. In fact, the estimates probably are conservative because people tend to live in concentrated areas, and these areas are more likely to be near schools. Because population distribution is even more varied in nonurban areas, no estimates were made of the population affected in these areas. Third, although the current SRTS legislation provides funds only for elementary and middle schools, high schools were included in the analysis. High schools may become eligible for SRTS funding in the future, and the increased awareness of the benefits of SRTS programs could lead to improvements in planning for high schools. Exclusion of the 15,478 high schools reduced the percentage of land within 0.5 mile of a school in large and small urban areas combined from 32% to 30%. Fourth, we used the most recent data on the number of schools, which was from 2003 through 2004, but the most recent U.S. Census population data available were from 2000. This may have led to underestimation of the population in rapidly growing areas. Finally, using a different radius for the school buffers would have yielded different results. However, even the half-mile buffers in some cases may include residences much farther than 1 mile from the school because of the road networks. A larger radius may be more appropriate when, for example, the target is bike riding. However, only 1.5% of children rode bikes to school in 2001 (14). Therefore, we focused on walking in small areas near schools.

### Conclusion

Our research provides estimates of the amount of land area and population in the United States that could be affected by SRTS programs, and it examines the types of locations where such improvements are likely to affect the greatest number of people. Most research conducted on SRTS improvements has focused on benefits to schoolchildren (15-17); this article estimates the effects that such improvements could have on the larger community. Communities with limited funds may be able to improve their overall walkability by using federal SRTS funding to improve walking and cycling routes to schools. Further research at the county or state level could examine more precisely how much of the overall community could benefit from proposed SRTS projects, and this information might be helpful in deciding which SRTS projects should get priority funding.

## Figures and Tables

**Table.** Characteristics of Land Areas in the United States Within 0.5 Mile of a Public School^a^, by Population Category

**Area**	No. Areas	Total Population, 2000	No. Public Schools	Area(Square Miles)	Area Within 0.5 Mile of Schools (Square Miles)	Percentage of Area Within 0.5 Mile of Schools
**Urban areas^b^**						
Large (population ≥1,000,000)	37	116,647,640	25,938	34,040	13,275	39.0
Small (population 50,000-999,999)	428	75,676,184	19,740	40,409	10,700	26.5
**Metropolitan areas outside urban areas^c^**	1088	55,846,061	16,142	843,240	9164	1.1
**Nonmetropolitan areas^c^**	2048	48,835,682	24,099	2,680,545	12,708	0.5
Total	3601	297,005,567	85,919	3,598,234	45,847	NA

NA indicates not applicable.

a School location data is from Spatial Insights, Inc (6).

b Urban areas (UAs) are defined by the U.S. Census Bureau as being densely settled territory containing more than 50,000 people, with core census block groups or blocks that have a population density of at least 1000 people per square mile, and with surrounding census blocks that have an overall density of at least 500 people per square mile (7).

c Areas outside of UAs were categorized as metropolitan or nonmetropolitan based on rural–urban continuum codes from the U.S. Department of Agriculture (9). Only the portions of metropolitan counties outside of UAs were counted as metropolitan areas; portions of counties within UAs were counted as UAs. Therefore, a metropolitan county may be divided in these analyses as a part urban area and a part metropolitan area (not urban).
